# Modes of invasion during tumour dissemination

**DOI:** 10.1002/1878-0261.12019

**Published:** 2016-12-09

**Authors:** Pahini Pandya, Jose L. Orgaz, Victoria Sanz‐Moreno

**Affiliations:** ^1^ Tumour Plasticity Team Randall Division of Cell and Molecular Biophysics King's College London UK

**Keywords:** actomyosin contractility, cancer metastasis, invasion, plasticity, Rho GTPases

## Abstract

Cancer cell migration and invasion underlie metastatic dissemination, one of the major problems in cancer. Tumour cells exhibit a striking variety of invasion strategies. Importantly, cancer cells can switch between invasion modes in order to cope with challenging environments. This ability to switch migratory modes or plasticity highlights the challenges behind antimetastasis therapy design. In this Review, we present current knowledge on different tumour invasion strategies, the determinants controlling plasticity and arising therapeutic opportunities. We propose that targeting master regulators controlling plasticity is needed to hinder tumour dissemination and metastasis.

**Abbreviations**
Cdc42: cell division cycle 42
ECM: extracellular matrix
EMT: Epithelial‐to‐mesenchymal transition
ERM: ezrin/radixin/moesin
ERULS: ezrin‐rich uropod‐like structure
ESCRT: endosomal sorting complexes required for transport
GAP: GTPase‐activating proteins
GEF: guanine exchange factor
LIMK: LIM kinase
MAT: mesenchymal‐to‐amoeboid transition
MLCK: myosin light chain kinase
MMP: matrix metalloproteinase
MRCK: myotonic dystrophy kinase‐related Cdc42‐binding kinase
MYPT1: myosin phosphatase target subunit‐1
NMII: nonmuscle myosin II
PAK: p21‐associated kinases
PIG3: p53‐induced gene 3 protein
Rac: Ras‐related C3 botulinum toxin substrate
Rho: Ras homolog family member
RLC: regulatory light chain
ROCK: Rho‐associated coiled‐coil‐containing protein kinase
ROS: reactive oxygen species
SDF‐1: stromal cell‐derived factor 1
uPA: urokinase plasminogen activator
uPAR: uPA receptor
ZIPK: zipper‐interacting protein kinase

## 1 Cancer cell invasion and dissemination

**Figure 1:**
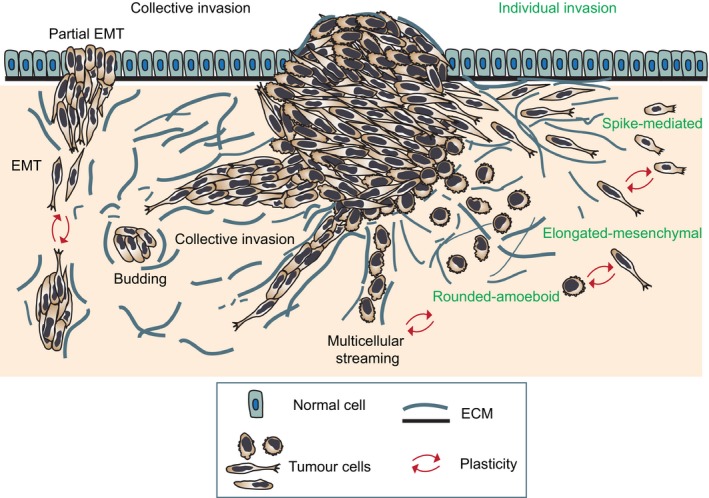
Modes of invasion during tumour dissemination. Diagram showing the main individual and collective modes of tumour invasion and plasticity that allows interconversion between modes. Cells invading individually can use protrusion‐based elongated‐mesenchymal, bleb‐ and contractility‐driven rounded‐amoeboid and filopodial spike‐mediated strategies. When cell–cell junctions are maintained, cells can move collectively as multicellular streams, budding or larger clusters (collective invasion). Migratory plasticity drives interconversion between the different modes.

Abnormal tumour cell migration and invasion underlies metastatic dissemination, a major clinical problem in cancer (Sanz‐Moreno and Marshall, 2010). Metastasis is a multistage process involving cell migration and invasion, transit in the blood or lymph, extravasation and colonization in the secondary site. Acquisition of invasive behaviour involves activation of signalling pathways controlling cytoskeletal dynamics, as well as turnover of cell–matrix and cell–cell adhesions (Fig. 1; Friedl and Alexander, 2011). Cancer invasion is a heterogeneous and adaptive process involving changes in cell morphology and generation of cell polarity, resulting in translocation of the cell body. Cancer cells display exceptional ability to adapt to different environmental conditions engaging in different migration strategies, as reviewed in Clark and Vignjevic (2015); Friedl and Alexander (2011); Sahai (2005). Cancer cells can migrate either individually in the absence of cell–cell junctions, or collectively upon retention of cell–cell adhesions (Friedl and Alexander, 2011; Fig. 1). In turn, cancer cells can use a number of strategies when migrating individually (elongated‐mesenchymal, rounded‐amoeboid, spike‐mediated) or collectively (multicellular streaming, tumour budding, collective invasion; Fig. 1). Studies using histopathological human samples and intravital imaging of xenografted tumours in mice have shown that these strategies can be observed *in vivo*, as reviewed in Clark and Vignjevic (2015); Friedl and Gilmour (2009); Friedl *et al*. (2012). While collective cell migration allows entry into the lymphatic system, individual cell migration is essential for entry into the bloodstream and dissemination to distant sites (Giampieri *et al*., 2009).

In this Review, we describe the different individual and collective modes of invasion, the plasticity that cancer cells display, enabling them to switch between different migratory modes and the determinants of this plasticity. We also discuss the therapeutic challenges arising from migratory plasticity that could explain failure of some therapies, and the potential targets that could lead to a complete blockade in cancer cell migration and invasion. We propose that targeting master regulators controlling plasticity is needed to hinder tumour dissemination and metastasis. While this Review tries to cover the different modes of migration and key aspects of migratory plasticity during invasion and metastasis, it is beyond the scope of this work to provide detailed insight into each section. Hence, throughout the Review, readers are directed to other excellent reviews that cover the relevant topic in depth.

## 2 Cell migration mechanisms

The molecular interactions between F‐actin and nonmuscle myosin II (NMII) govern the generation of mechanical forces across diverse length scales, and these are important not only for migration (Murrell *et al*., 2015; Vicente‐Manzanares *et al*., 2009) but also for modulating cytokinesis (Green *et al*., 2012) and tissue morphogenesis (Murrell *et al*., 2015; Salbreux *et al*., 2012).

During cell migration (Fig. 1), directional polarity is achieved by cells generating a leading edge at the front and a lagging edge at the back (reviewed in Ridley, 2015). Protrusion and adhesion of the leading edge and retraction of the rear edge drive movement in the direction of locomotion (Richardson and Lehmann, 2010). The dynamics of cytoskeletal coupling with cell surface receptors that engage with surrounding tissue structures is the key process underlying all forms of migration (Friedl and Alexander, 2011).

Cell migration is a cyclic process (Friedl and Wolf, 2009; Lauffenburger and Horwitz, 1996) that begins with actin polymerization on one side of the cell resulting in actin‐rich protrusion at the leading edge. Migration is facilitated by the forward movement of the cell, which is achieved by the engagement of cell surface receptors with the extracellular matrix (ECM); the formation of leading edge adhesions associated with proteolytic degradation of the ECM; and actomyosin contractility‐mediated retraction of the rear edge of the cell.

Actin polymerization and organization into different cytoskeletal structures is regulated by the Rho family of proteins that play a central role in cell migration and has been extensively reviewed in Ridley (2015). Rho GTPases are molecular switches that cycle between active states when bound to GTP and inactive states when bound to GDP. This is regulated by activators or guanine exchange factors (GEFs) and inactivators or GTPase‐activating proteins (GAPs; Ridley, 2015). By interacting with specific downstream effectors, active GTPases induce diverse actin rearrangements (Heasman and Ridley, 2008).

Three prototypical members of the family, Ras‐related C3 botulinum toxin substrate (Rac), Ras homolog family member (Rho) and cell division cycle 42 (Cdc42), have been extensively linked to cell migration regulation (Ridley, 2015). Rho induces unbranched actin polymerization via formin mDia1, while Rho‐associated coiled‐coil‐containing protein kinase (ROCK) promotes bundling of actomyosin filaments resulting in either stress fibres or an actomyosin cortex (Kimura *et al*., 1996; Otomo *et al*., 2005). Activation of ROCK downstream of Rho results in activating phosphorylation of myosin II (Amano *et al*., 1996) and inactivation of myosin phosphatase target subunit‐1 (MYPT1; Kimura *et al*., 1996). Phosphorylated myosin II drives contraction of actin fibres in an ATP‐dependent manner (Scholey *et al*., 1980; Wang *et al*., 2003). In addition to myosin II, ROCK can also phosphorylate ezrin/radixin/moesin (ERM), LIM kinases (LIMK1, LIMK2), α‐adducin and several other proteins important for migration (Kimura *et al*., 1996; Matsui *et al*., 1998; Ohashi *et al*., 2000).

Rac and Cdc42 also regulate actin polymerization (Ridley *et al*., 2003; Wojciak‐Stothard and Leiper, 2008). Binding of Cdc42 to myotonic dystrophy kinase‐related Cdc42‐binding kinase (MRCK) results in phosphorylation of myosin II, MYPT1, LIMK1, LIMK2 and moesin (Leung *et al*., 1998; Nakamura *et al*., 2000; Scott and Olson, 2007; Tan *et al*., 2001b). The activation of LIM kinases by phosphorylation allows for the inactivating phosphorylation of actin‐severing protein cofilin, which inhibits actin depolymerization (Maekawa *et al*., 1999; Sumi *et al*., 1999). Rac proteins interact with lamellipodin and the WAVE complex that, in turn, promote actin nucleation by the Arp2/3 complex (Law *et al*., 2013; Ridley, 2015).

Another set of downstream effectors of Rac and Cdc42 include the p21‐associated kinases (PAKs). PAK1 promotes motility by inducing rapid turnover of focal contacts at leading edge of cells via phosphorylation of paxillin (Brown *et al*., 2002; Nayal *et al*., 2006; Premont *et al*., 2004). PAK‐mediated actin remodelling also involves LIMK1 (Edwards *et al*., 1999; Yang *et al*., 1998).

Actin polymerization by Rho GTPases directs the forces generated by actomyosin contractility needed for migration to take place. F‐actin polymers serve as the scaffold for myosin II motors and accessory proteins (Murrell *et al*., 2015; Vicente‐Manzanares *et al*., 2009) that can walk along, propel the sliding of or produce tension on actin filaments via ATPase activity (Vicente‐Manzanares *et al*., 2009). Depending on the location of myosin with respect to the middle filaments, this can result in the contraction or extension of two bound actin filaments. The contractile activity of NMII can be regulated via reversible phosphorylation of Ser19 on the regulatory light chain (RLC; Hirata *et al*., 2009) by ROCK, myosin light chain kinase (MLCK) and other kinases such as MRCK, citron kinase, LIMK, zipper‐interacting protein kinase (ZIP kinase) and Ca^2+^/calmodulin‐dependent protein (Endo *et al*., 2004; Kimura *et al*., 1996; Madaule *et al*., 1998; Poperechnaya *et al*., 2000; Tan *et al*., 2001a). Subsequent phosphorylation at Thr18 of the RLC further increases the contractile activity of myosin II (Hirata *et al*., 2009; Umemoto *et al*., 1989). For detailed function of myosin II, readers are referred to the review (Vicente‐Manzanares *et al*., 2009).

## 3 Collective modes of cancer invasion

While the models above tend to focus on cells migrating as separate entities, cancer cell invasion is not restricted to cells moving individually. Histopathological samples show invasion of normal tissue by compact groups or clusters of cells and strands or cords of connected tumour cells (Clark and Vignjevic, 2015; Friedl and Gilmour, 2009; Friedl *et al*., 2012; Leighton *et al*., 1960; Wang *et al*., 2016; Willis, 1952). Likewise, intravital microscopy and *in vitro* studies have shown that cancer cells can move as loosely/nonadherent ‘streams’ of cells or collective migration of cell strands and sheets (Alexander *et al*., 2008; Clark and Vignjevic, 2015; Friedl *et al*., 2012). At the invasive front (tumour border) of certain cancer types, such as some carcinomas, invasive cells are observed to migrate as collective groups (Christiansen and Rajasekaran, 2006; Friedl *et al*., 1995, 2004). Furthermore, collective cancer invasion can be seen as a dysregulated recapitulation of key steps that occur in many physiological processes such as embryonic morphogenesis or regeneration and tissue repair after wounding (Friedl and Gilmour, 2009).

Transition from collective to single‐cell invasion may enhance metastatic efficiency and has been reviewed in Friedl *et al*. (2012). However, intravasation into lymphatic vessels can be efficiently performed by cell groups or clusters (Byers *et al*., 1995; Giampieri *et al*., 2009; Hashizume *et al*., 1996; Madhavan *et al*., 2001). This is also supported by the existence of circulating tumour clusters from patient peripheral blood samples (Aceto *et al*., 2014; Brandt *et al*., 1996; Hart, 2009; Hou *et al*., 2011; Kats‐Ugurlu *et al*., 2009; Khoja *et al*., 2014).

Similar to single‐cell migration, collective cell movement results from the coordinated actions of the actin cytoskeleton, actomyosin contraction, cell polarity and cell surface receptors that engage with surrounding tissue structures (Friedl and Alexander, 2011; Ridley *et al*., 2003). While collective cell migration also follows the cyclical process described above for single‐cell migration (Friedl and Wolf, 2009; Lauffenburger and Horwitz, 1996), in collective movement cells remain grouped by cell–cell junctions (Friedl *et al*., 2004, 2012; Rorth, 2007). Protrusion extension and retraction are coordinated in a ‘supracellular manner’, in which cytoskeletal protrusion and contractility are mechanically mediated through cell–cell junctions and involve several cells (Friedl *et al*., 1995; Hegerfeldt *et al*., 2002; Hidalgo‐Carcedo *et al*., 2011; Tambe *et al*., 2011). Therefore, collective cell migration involves coordinating cell movement with ‘supracellular’ polarity, cytoskeletal organization and cell–cell junction stability (Friedl and Gilmour, 2009; Friedl *et al*., 2012).

Both histopathological studies of cancer tissues and those using intravital microscopy have shown distinct modes of collective cancer migration (Fig. 1), as reviewed in Clark and Vignjevic (2015); Friedl and Gilmour (2009); Friedl *et al*. (2012). These sometimes overlapping strategies are determined by a combination of parameters such as degree of cell–cell adhesion, cellular morphology and supracellular coupling of cell–cell signalling (Friedl *et al*., 2012).

### 3.1 Multicellular streaming

During multicellular streaming, cells move one after the other in the same path within the tissue (Fig. 1; Friedl *et al*., 2012; Friedl and Wolf, 2003; Manning *et al*., 2015). In this migratory mode, cells are typically guided by chemokine or morphogen gradients or ECM structures (i.e. ‘microtracks’; Friedl *et al*., 1997; Haeger *et al*., 2015). Hence, coordinated migration takes place as directed movement of small strands of single cells, multicellular streams and as diffuse infiltration (‘chain‐ or swarm‐like’; Friedl and Alexander, 2011; Kedrin *et al*., 2008; Patsialou *et al*., 2013; Roussos *et al*., 2011; Seftor *et al*., 2002; Wyckoff *et al*., 2004). These chains (‘Indian files’) have been observed in infiltrating breast carcinoma (Page and Anderson, 1987; Pitts *et al*., 1991), ovarian cancer (Sood *et al*., 2001) and melanoma (Friedl and Wolf, 2008; Seftor *et al*., 2002). Importantly, in this mode of migration, each cells’ cytoskeleton acts independently to generate traction force on the matrix, while cell–cell adhesions are weak or short‐lived (Friedl *et al*., 2012), allowing velocities similar to those achieved by cells migrating individually (1–2 μm·min^−1^ or even faster; Clark and Vignjevic, 2015; Friedl *et al*., 2012). Streaming cells can display rounded‐amoeboid or elongated‐mesenchymal phenotypes (Clark and Vignjevic, 2015; Friedl and Alexander, 2011; Friedl *et al*., 2012). Intravital studies have shown that cells that display rounded‐amoeboid morphology *in vitro*, such as human and mouse melanoma cells, are more likely to migrate as single cells or as streams *in vivo* (Herraiz *et al*., 2016; Manning *et al*., 2015; Pinner and Sahai, 2008a,b; Sanz‐Moreno *et al*., 2008).

### 3.2 Tumour budding

Scattered clusters of approximately five cells (‘tumour buds’) located in close proximity ahead of the invasive front (Fig. 1) have also been observed in colorectal cancer (Brabletz *et al*., 2001; Bronsert *et al*., 2014; Carr *et al*., 1986; Prall *et al*., 2005) and carcinomas from the oesophagus, pancreas, lung and breast (reviewed in Grigore *et al*., 2016). Studies using 3D reconstructions from 2D serial sections of colorectal (Carr *et al*., 1986) and other cancer types (pancreatic, lung, breast; Bronsert *et al*., 2014) demonstrated that tumour budding is a dynamic process by which the tumour mass extends several finger‐like multicellular projections that, later, break away from the main tumour mass as small cell clusters (tumour buds; Bronsert *et al*., 2014; Carr *et al*., 1986). Importantly, tumour budding has been associated with poor cancer outcomes (Grigore *et al*., 2016).

### 3.3 Collective cell invasion

This mode involves compact and cohesive cell groups with two or more neighbouring cells (Fig. 1). Collective invasion is facilitated by long‐lived cell–cell junctions (Alexander *et al*., 2008; Friedl *et al*., 1995, 2012). Cells may adopt different morphologies depending on cell type and number and the structure of the tissue invaded (Friedl and Alexander, 2011).

These groups can be composed of small clusters, solid strands or files (1–2 cells in diameter) up to broad masses (Wolf *et al*., 2007) that can even form an inner lumen if epithelial polarity is maintained, as seen in some breast, prostate, pancreatic and colorectal tumours (Christiansen and Rajasekaran, 2006; Friedl and Gilmour, 2009; Friedl *et al*., 2012; Nabeshima *et al*., 1999). Protruding sheets and strands that remain in contact with the primary site and generate local invasion have been detected in invasive epithelial tumours such as oral squamous cell carcinoma and mammary carcinoma (Bell and Waizbard, 1986; Page and Anderson, 1987), colon carcinoma (Nabeshima *et al*., 1999), basal cell carcinoma and others (Friedl and Wolf, 2003). Cell clusters or ‘nests’ that detach from the primary tumour and extend into surrounding tissue have been described in epithelial cancers, melanoma and rhabdomyosarcoma (Ackerman and Ragaz, 1984; Bell and Waizbard, 1986; Nabeshima *et al*., 1999; Page and Anderson, 1987).

**Figure 2:**
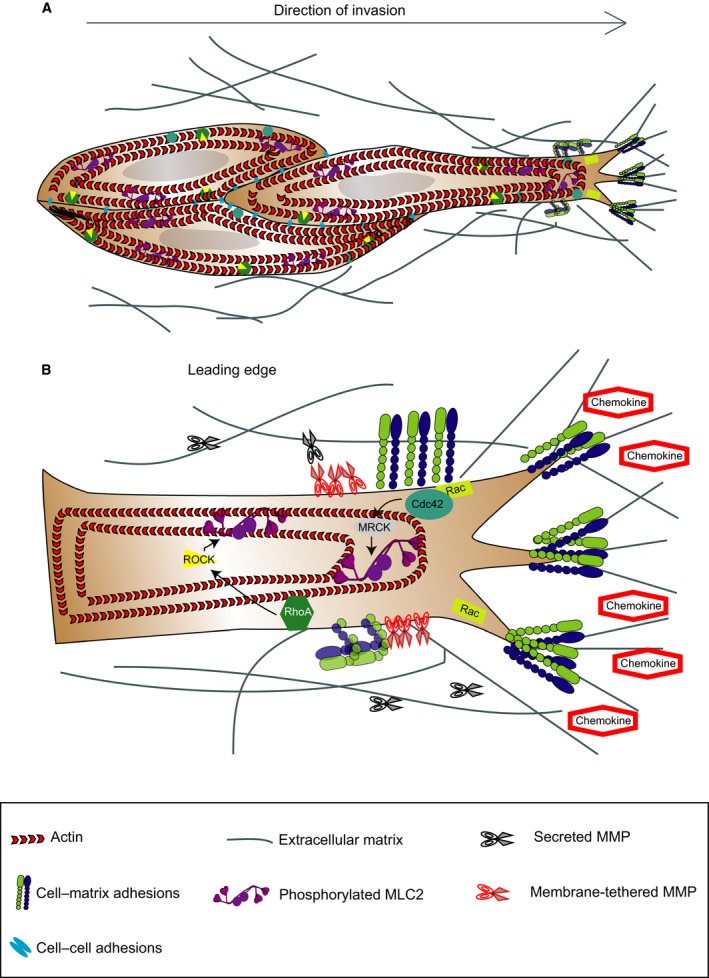
Signalling pathways controlling collective modes of invasion. (A) Diagram showing the key regulators of collective migration. The leading edge of the multicellular group comprises one (or several) leader cells with mesenchymal characteristics. Leader cells extend actomyosin‐mediated actin‐rich protrusions that generate integrin‐mediated forward traction and pericellular proteolysis yielding a re‐aligned ECM that guides the group. Following cells are passively dragged behind along the established migration track by cell–cell adhesion. (B) Diagram showing the intracellular pathways activated in response to external stimuli and proteolysis of ECM. Membrane receptors such as β1 integrins control migration of individual elongated‐mesenchymal cells. Rac activation at the leading edge allows for protrusion formation that is linked to a ‘supracellular’ cytoskeleton. Activation of myosin II‐based contractile forces by Rho‐ROCK and Cdc42‐MRCK signalling allows for contraction of cell body and retraction of the rear.

In the most cases, the leading edge of the multicellular group is composed of one or several leader cells with mesenchymal characteristics (Fig. 1). Leader cells extend actomyosin‐mediated actin‐rich protrusions that generate integrin‐mediated forward traction (Hegerfeldt *et al*., 2002) and pericellular proteolysis towards the tissue structure (Nabeshima *et al*., 2000; Wolf *et al*., 2007), which yields a re‐aligned ECM that guides the group (Fig. 2A; Gaggioli *et al*., 2007; Khalil and Friedl, 2010). Following cells are passively dragged behind along the established migration track by cell–cell adhesion (Fig. 2A; Friedl *et al*., 1995). Nevertheless, follower cells reinforce this ECM alignment and increase the diameter of the invading strand (Friedl and Wolf, 2008).

From studies using intravital microscopy, breast cancer cells or fibrosarcoma cells with predominantly individual phenotypes (Alexander *et al*., 2008; Giampieri *et al*., 2009; Roussos *et al*., 2011) are more prone to switching between single‐cell and collective migration modes (see ‘Plasticity during collective invasion’ section). Collective invasion is typically the slowest migratory mode (0.01–0.05 μm·min^−1^; Weigelin *et al*., 2012). Looking for advantages of this slower mode of migration, it has been suggested that the large cell mass could secrete high concentrations of promigratory factors and matrix proteases and protect inner cells from immune clearing. In addition, more migratory clones within the group could promote invasion of less motile cells, thereby increasing overall tumour invasion (Friedl and Wolf, 2003).

## 4 Plasticity during collective invasion

Extrinsic and intrinsic factors determine the adaptation of tumour cells to modify their migration mechanism (Friedl, 2004; Friedl and Alexander, 2011). This adaptive, dynamic behaviour is termed plasticity of tumour cell migration, and it is a combination of specific morphologic and mechanistic entities (Fig. 1). However, cells often display heterogeneity and can exhibit multiple modes of migration in 3D tissues (Fig. 1; Friedl and Wolf, 2010; Wolf *et al*., 2003). Furthermore, some cancer cells can spontaneously switch between different modes of migration (Sanz‐Moreno *et al*., 2008).

Extensive research has been performed in the last 15 years trying to understand the mechanisms supporting different types of migration and the signals and conditions that trigger tumour cell plasticity (Friedl and Alexander, 2011; Lauffenburger and Horwitz, 1996; Ridley *et al*., 2003; Sanz‐Moreno and Marshall, 2010). By understanding this complex array of extracellular and intracellular determinants, the general machinery governing most types of cancer migration could be identified holding promise to translation into therapeutic interventions.

### 4.1 Epithelial‐to‐mesenchymal transition (EMT) and partial EMT

In epithelial cancers, EMT is a molecular programme characterized by loss or weakening of cell–cell junctions, which disrupts apico‐basal polarity and cell anchoring to the basement membrane (Thiery *et al*., 2009). This leads, in turn, to individual cell migration with enhanced migratory and invasive capacity, increased resistance to apoptosis and augmented ECM production (Kalluri, 2009). EMT can be complete or partial depending on the degree of cell–cell adhesion (Fig. 1). Therefore, EMT‐like dissemination without the typical EMT‐associated gene expression patterns has been observed (Christiansen and Rajasekaran, 2006; Gavert *et al*., 2011; Wicki *et al*., 2006). Colorectal carcinomas often display cohesive cells at the leading edge, small groups of cells and individual cells scattered without connection to the main tumour, indicative of different degrees of EMT (Brabletz *et al*., 2001; Gavert *et al*., 2007).

### 4.2 Collective‐to‐individual transition

When cell–cell and cell–ECM interactions are simultaneously weakened, a transition from collective invasion to single‐cell migration takes place (Fig. 1; Friedl, 2004). In multicellular clusters invading away from melanoma explants, the inhibition of β1 integrin by blocking antibodies abolishes collective movement by inducing the detachment of individual cells (Hegerfeldt *et al*., 2002). This mechanism could involve an intermediate mesenchymal migration step that would later lead to rounded‐amoeboid single‐cell dissemination (Friedl, 2004). Collective invasion from fibrosarcoma and breast carcinoma spheroids can be abolished by proteolytic inhibition or by collagenase MT1‐MMP knock‐down, leading to nonproteolytic single‐cell dissemination (Wolf *et al*., 2007).

### 4.3 Determinants of plasticity

The ability to switch between various modes of migration is regulated by signalling pathways and sustained via transcriptional programmes. This, in turn, can facilitate efficient invasion and distant metastasis by conferring increased resistance to external stimuli and adaptability to different microenvironments. Plasticity requires integration of intracellular and extracellular physical and molecular cues (Friedl, 2004; Salbreux *et al*., 2012). In this section, we describe how cancer cells translate extracellular signals into intracellular responses that impact the mode of migration.

Factors determining plasticity during collective migration include physical cues and molecular cues (Fig. 2).

#### 4.3.1 Physical cues

The molecular and physical characteristics of the ECM, such as composition, geometry, porosity, alignment and stiffness, strongly contribute to cell adhesion, migration and invasion (Wolf and Friedl, 2011). As such, pericellular proteolysis generated by tumour‐ and stromal cell‐derived proteases generates micro‐ and macrotracks (micro‐ and macropatterning, respectively; Friedl and Wolf, 2008) surrounded by collagen bundles that support collective invasion (Friedl *et al*., 1997; Gaggioli *et al*., 2007; Wolf *et al*., 2007). In addition, force‐mediated ECM remodelling favours collective breast carcinoma cell invasion (Provenzano *et al*., 2008; Fig. 2A). Mechanical cues affecting modes of cell migration include confinement and topology, among other factors (Kurniawan *et al*., 2016).

#### 4.3.2 Molecular cues

##### 4.3.2.1 Proteases

Tumour invasion and progression have been linked to upregulation of proteases (Egeblad and Werb, 2002; Wolf and Friedl, 2011) with highest levels of activated proteases expressed at the tumour–stromal interface (Sternlicht *et al*., 2000). These proteases include matrix metalloproteinases (MMPs), ADAMs, cathepsins, urokinase plasminogen activator (uPA) and its receptor uPAR (Mason and Joyce, 2011; Rizki *et al*., 2008). Proteases contribute towards ECM degradation and tissue remodelling to form ECM bundles as well as generation of active epitopes of ECM components (Gaggioli *et al*., 2007; Kenny *et al*., 2008). The localized cleavage of ECM fibres by proteases results in release of ECM‐imposed confinement, allowing the relaxation of the nucleus and enhancing migration speeds (Wolf *et al*., 2007, 2013). As a consequence, the degree of proteolytic cleavage of ECM determines the degree of deformation and the confinement experienced by the cell.

During collective migration, cells at the leading edge of collectively invading colorectal carcinomas show increased expression and activity of membrane‐tethered MT1‐MMP and secreted MMP2, leading to polarized ECM degradation (Nabeshima *et al*., 2000; Fig. 2B). MT1‐MMP is essential in collagen processing and multicellular strand formation during collective invasion of fibrosarcoma cells (Wolf *et al*., 2007).

##### 4.3.2.2 Membrane receptors

Extracellular matrix‐binding molecules also determine the mode of invasion. Integrins couple the ECM to the actin cytoskeleton and develop small focal complexes (Friedl and Wolf, 2003; Hynes, 2002), which allow Rho GTPase‐mediated outside‐in signalling (Geiger and Peeper, 2009; Grashoff *et al*., 2010; Hodivala‐Dilke *et al*., 1999; Lee *et al*., 2009; Ridley *et al*., 2003; Fig. 2B).

β1 Integrins can control migration of multicellular melanoma (Hegerfeldt *et al*., 2002) and ovarian carcinoma (Casey *et al*., 2001).

CD44 binds to different ECM proteins (Zoller, 2011) and connects to the actin cytoskeleton through the ERM complex and ankyrin, signalling also through Rho GTPases (Zoller, 2011). CD44 serves also as a co‐receptor for other adhesion molecules such as integrins and podoplanin; the latter signals to enhance RhoA activity, increasing collective invasion of squamous cell carcinomas (Martin‐Villar *et al*., 2006).

DDR family of receptors interact with fibrillar collagen and signal through several intracellular pathways (STAT5, NF‐kB, p38 MAPK/ERK and Src‐family kinases; Neuhaus *et al*., 2011; Vogel *et al*., 2006). When co‐engaged with DDR1, E‐cadherin signalling limits excessive actomyosin contractility along cell–cell junctions; this stabilizes junctions and, in turn, maintains collective invasion (Hidalgo‐Carcedo *et al*., 2011).

In addition to cell–matrix adhesion, collective migration is also enabled by cell–cell adhesions through different adhesion systems, such as cadherins, tight junctions, gap junctions and others (Friedl *et al*., 2012; Hegerfeldt *et al*., 2002; Hidalgo‐Carcedo *et al*., 2011; Fig. 2A). Loss or downregulation of E‐cadherin expression that drives EMT seems to be tunable, therefore leading to complete or partial EMT. In the latter, different E‐cadherin levels that do not confound migration may be retained, or alternative proinvasive cadherins including N‐ or VE‐cadherin may be expressed (Yano *et al*., 2004). Collective invasion with E‐cadherin in cell–cell junctions can be facilitated upon upregulation of L1‐CAM (Gavert *et al*., 2011; Shtutman *et al*., 2006) and upregulation of podoplanin, which activates RhoA (Wicki *et al*., 2006).

##### 4.3.2.3 Secreted factors

Extracellular chemokines, cytokines and growth factors secreted by tumour or stromal cells enable and promote migration in a paracrine and autocrine fashion (Friedl and Alexander, 2011; Haeger *et al*., 2015). In addition, ECM degradation allows the release of these factors that can also be processed by proteases resulting in their activation, inactivation or degradation (Dean *et al*., 2008; Mu *et al*., 2002; Shiao and Coussens, 2010; Sounni *et al*., 2010).

Invasion‐promoting chemokines, growth factors and their receptors engage intracellular signalling networks (JAK, PI3K, Src, ERK) and/or Rho GTPase activity (Friedl and Alexander, 2011; Fig. 2B). Collective invasion of oral squamous carcinoma cells is stimulated by stromal cell‐derived factor 1 (SDF‐1) and HGF secreted from stromal fibroblasts in response to tumour‐derived IL‐1α (Daly *et al*., 2008). Likewise, a paracrine loop between tumour‐associated macrophages secreting EGF and breast carcinoma cells secreting CSF‐1 drives cancer cell migration (Wyckoff *et al*., 2004).

##### 4.3.2.4 Intracellular signalling pathways

Effective collective migration requires supracellular coordination of the cytoskeleton, which is controlled by Rho GTPase signalling (Friedl and Alexander, 2011). Leader cells generate actomyosin‐ and integrin‐mediated traction towards the ECM, controlling tensional regulation of ECM alignment (Hegerfeldt *et al*., 2002). High Cdc42/MRCK‐ and ROCK‐mediated actomyosin contractility levels are found at the edges of groups of invading cancer cells (Gaggioli *et al*., 2007; Fig. 2B). Actomyosin contractility generates pulling forces between the substrate and the follower cells, which, together with cortical actomyosin at lateral regions of the groups, maintain coupling between cells and collective forward movement in melanoma (Hegerfeldt *et al*., 2002) and squamous cell carcinoma (Gaggioli *et al*., 2007; Hidalgo‐Carcedo *et al*., 2011). Cell contractility mediated by Rho/ROCK/MLCK is also required for retraction of the tail in migrating groups and for lateral mechanocoupling via cadherin‐based adhesions (Vicente‐Manzanares *et al*., 2009).

## 5 Individual cancer cell invasion

Cancer cells can also invade individually in the absence of cell–cell junctions using a variety of strategies (Fig. 1).

### 5.1 Elongated‐mesenchymal mode of invasion

**Figure 3:**
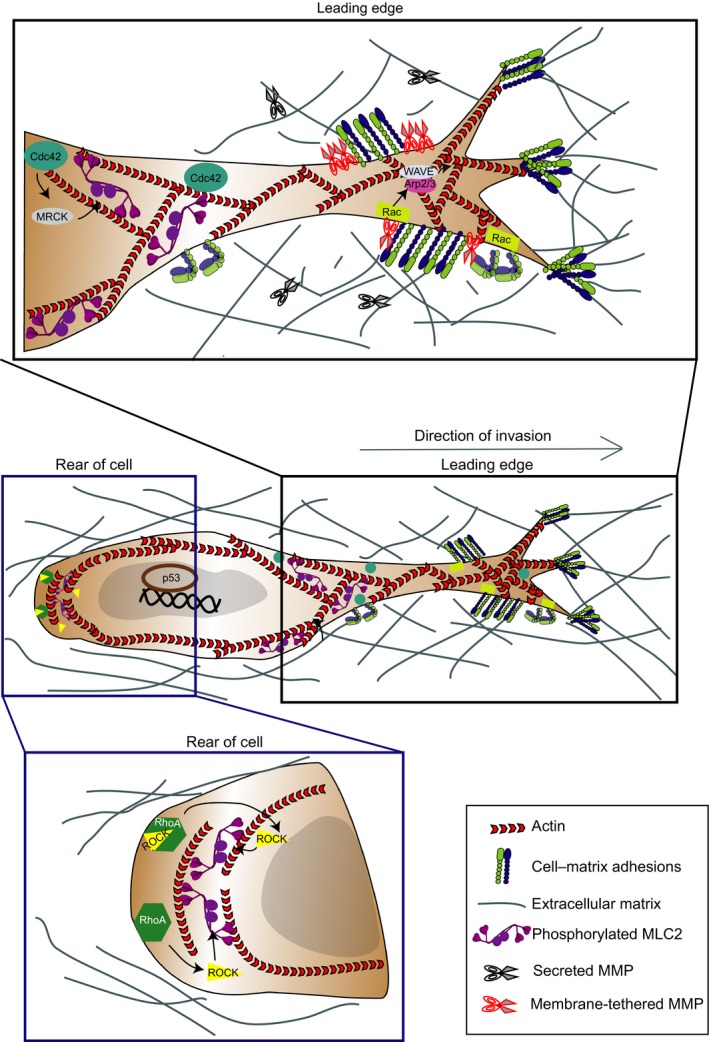
Signalling pathways controlling elongated‐mesenchymal mode of invasion. Diagram showing key regulators of elongated‐mesenchymal mode of migration in cells. During this mode, cells adopt an elongated morphology that is characterized by actin‐rich protrusions, focal adhesion formation, matrix metalloproteinase (MMP) activity and actomyosin contractility localized at the rear of the cells. Top inset: signalling activity at the leading edge of cells exhibiting elongated‐mesenchymal migration. Polarized signalling of GTPase Rac1 directs Arp2/3 via WAVE2 to drive actin polymerization in branched filaments against the plasma membrane. Bottom inset: signalling activity at the rear of cells exhibiting elongated‐mesenchymal migration. Rho‐ROCK signalling is required for the contractile activity of actomyosin scaffold to retract the cell rear. Transcription driven by p53 promotes elongated‐mesenchymal strategies.

On stiff 2D matrices and 3D matrices such as collagen I, adherent cancer cells arising from connective tissues, such as sarcomas, gliomas and some epithelial cancers (Paulus *et al*., 1996; Polette *et al*., 1998; Wolf *et al*., 2003), can adopt actin‐rich protrusions for migration. During this mode of migration, cells have an elongated morphology (Fig. 3) that is characterized by focal adhesion formation, MMP activity and actomyosin contractility localized at the rear of the cells. The requirement of strong focal adhesion limits velocity for cells adopting elongated‐mesenchymal mode of migration resulting in relatively slow speed (0.1–2 μm·min^−1^ *in vitro*; Friedl, 2004).

Elongated‐mesenchymal migration is a protrusion‐dependent mode mediated by polarized signalling of GTPases Rac1 (Sanz‐Moreno *et al*., 2008; Yamazaki *et al*., 2009) and Cdc42 (Nalbant *et al*., 2004), which direct Arp2/3 to drive actin polymerization in branched filaments against the plasma membrane (Amann and Pollard, 2001; Giri *et al*., 2013; Machesky *et al*., 1999). Adhesion maturation is controlled by signalling activity of RhoA and effector proteins such as formin protein diaphanous homologs 1 and 2, while Rho‐ROCK signalling is required for the contractile activity of actomyosin scaffold to retract the cell rear (Friedl and Wolf, 2009; Ridley *et al*., 2003; Fig. 3).

### 5.2 Rounded‐amoeboid mode of invasion

**Figure 4:**
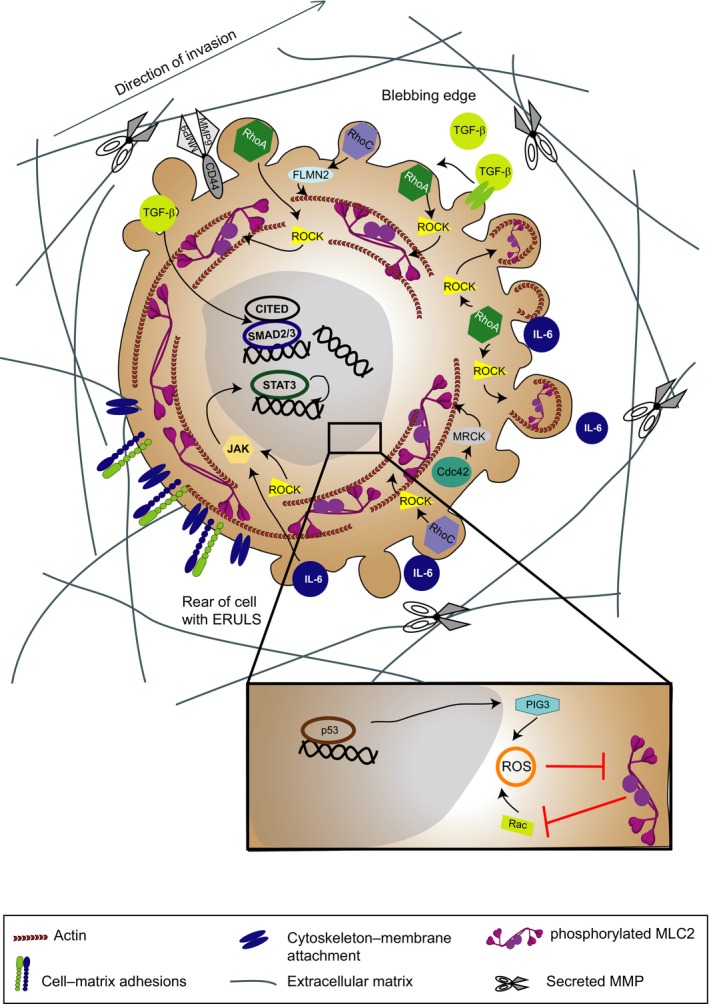
Signalling pathways controlling rounded‐amoeboid mode of invasion. Diagram showing key regulators of rounded‐amoeboid mode of migration. Rounded‐amoeboid cells squeeze through the matrix using small, unstable blebs present throughout the surface of the cells except at the rear, due to the presence of ezrin‐rich uropod‐like structures (ERULS) that determine polarity. Blebs are a consequence of low membrane–cortex attachment, increased intracellular pressure, high actomyosin contractility, low degree of β1 integrin‐mediated adhesion, reduced focal adhesion size and force generation. Rounded‐amoeboid motility is supported by high levels of actomyosin contractility downstream of Rho‐ROCK. While there is significant overlap in the RhoA‐ and RhoC‐mediated activation of actomyosin contractility, the assembly of cortical actin as a consequence of formin FLMN2 activation seems to be specific to RhoC. Maintenance of rounded‐amoeboid movement is driven by IL‐6 family of cytokines and the transcription factor STAT3. Conversely, ROCK can activate JAK/STAT3 signalling generating a positive feedback loop. TGF‐β promotes rounded‐amoeboid migration, which is perpetuated via SMAD2/CITED1‐mediated transcription. In addition, Rho/ROCK suppresses p53/PIG3‐mediated ROS production. On the other hand, Rac suppresses actomyosin contractility via ROS generation.

Cancer cells migrating across pliable matrices can use rounded‐amoeboid strategies and squeeze through the matrix using small, unstable blebs present throughout the surface of the cell (Sahai and Marshall, 2003; Sanz‐Moreno and Marshall, 2010; Sanz‐Moreno *et al*., 2008) except at the rear, due to the presence of ezrin‐rich uropod‐like structures (ERULS; Lorentzen *et al*., 2011) that dictate cell polarity (Fig. 4). Blebs are a consequence of low membrane–cortex attachment, increased intracellular pressure, low degree of β1 integrin‐mediated adhesion, reduced focal adhesion size and force generation (Bergert *et al*., 2015; Charras and Paluch, 2008; Charras and Sahai, 2014; Petrie *et al*., 2012; Sahai and Marshall, 2003; Sanz‐Moreno *et al*., 2008; Wolf *et al*., 2003). Due to low reliance on focal adhesions and their deformability, the average speed during rounded‐amoeboid migration can be significantly faster (2–25 μm·min^−1^ *in vitro*, 1–15 μm·min^−1^ *in vivo*) than the mesenchymal type of cell migration (Pankova *et al*., 2010; Pinner and Sahai, 2008a; Sanz‐Moreno *et al*., 2008).

Rounded‐amoeboid motility is supported by high levels of actomyosin contractility downstream of Rho‐ROCK (Sahai and Marshall, 2003; Sanz‐Moreno *et al*., 2008; Wilkinson *et al*., 2005; Yamazaki *et al*., 2009). There is significant overlap in the RhoA‐ and RhoC‐mediated activation of actomyosin contractility. Nevertheless, the assembly of cortical actin as a consequence of formin FLMN2 activation seems to be specific to RhoC in rounded‐amoeboid cells (Kitzing *et al*., 2010). Furthermore, mDia2–Dip interaction induces the characteristic cell blebbing in rounded‐amoeboid movement (Eisenmann *et al*., 2007).

In rounded‐amoeboid migrating cells, a local decrease in attachment of the cell membrane to the actin cortex or local rupture of the actin cortex initiates a cycle of bleb expansion and retraction that allows cell movement (Charras and Paluch, 2008). Bleb expansion appears to be a direct mechanical consequence of intracellular pressure pushing the membrane outwards in the direction of motion. Bleb expansion is then slowed down and inhibited by recruitment of membrane–cortex linker proteins that facilitate actin recruitment underneath the membrane. The retraction phase begins with rapid assembly of actomyosin filaments beneath the bleb membrane (Charras and Paluch, 2008). Bleb‐based movement is generated by creating blebs at the leading edge and exerting force onto the substrate to translocate the cell body (Charras and Paluch, 2008). These forces could be achieved by weakly adhering to the ECM or to surrounding cells; by applying forces on the ECM perpendicular to the direction of movement; or through nonspecific substrate friction (Bergert *et al*., 2015; Charras and Paluch, 2008). Tumour xenograft intravital imaging studies have shown that melanoma and breast cancer cells in the invasive fronts predominantly move using rounded‐amoeboid strategies (Giampieri *et al*., 2009; Herraiz *et al*., 2016; Pinner and Sahai, 2008a,b; Sanz‐Moreno *et al*., 2008, 2011). Importantly, the invasive fronts of human melanoma primary tumours and metastases are enriched in rounded cells (Cantelli *et al*., 2015; Orgaz *et al*., 2014; Sanz‐Moreno *et al*., 2011).

### 5.3 Other modes of individual invasion

While elongated‐mesenchymal and rounded‐amoeboid modes of migration are extremes of the spectrum, intermediate modes of migration have been reported as cells transition between these modes (Yin *et al*., 2013). Glioblastoma‐initiating cells can efficiently invade exhibiting a round cell body aided by long or short protrusions (Ruiz‐Ontanon *et al*., 2013). Under confinement, breast cancer cells exhibit a mode of migration that is dependent on directed water permeation. This mode, termed the osmotic engine model, relies on aquaporin5 and Na^+^/H^+^ exchangers (Stroka *et al*., 2014).

Another mode of migration described in the recent years is the lobopodial mode of migration. This pressure‐based mode involves the use of the nucleus as a piston to generate intracellular pressure that drives forward a blunt cylindrical protrusion termed lobopodia (Petrie *et al*., 2012, 2014). This mode is characterized by nonpolarized distribution of active Rac1 at the plasma membrane and RhoA‐driven actomyosin contractility at the front of the nucleus. Actomyosin contraction pulls the nucleus towards the front, which poses a diffusion barrier and results in increased intracellular pressure that pushes the leading edge forwards (Petrie *et al*., 2012, 2014). However, this mode of migration has only been described in fibroblasts and its role in cancer cell invasion remains to be established.

Furthermore, filopodial spike‐based cancer cell invasion has also been recently described (Paul *et al*., 2015; Fig. 1). In this mode, α5β1 integrin recycling promotes RhoA‐ROCK‐FHOD3‐driven invasion independently of Arp2/3 activity.

## 6 Plasticity during individual cell invasion

### 6.1 Mesenchymal‐amoeboid plasticity

As noted earlier, ECM degradation and tissue remodelling by secreted proteases regulate invasion (Friedl and Alexander, 2011; Mantovani *et al*., 2008). Importantly, pioneer work in the cell migration field showed that upon inhibition of pericellular proteases, elongated‐mesenchymal cells still invaded as rounded‐amoeboid cells both *in vitro* and *in vivo* (Sahai and Marshall, 2003; Wolf *et al*., 2003; Wyckoff *et al*., 2006) while undergoing mesenchymal‐to‐amoeboid transition (MAT; Friedl, 2004; Wolf *et al*., 2003). This plasticity most likely contributes to the failure of therapies targeting proteases (see section ‘Therapeutic challenges posed by migratory plasticity’).

As both actin assembly and the actomyosin machinery can regulate cell morphology, modulation of actin organization can predict the type of protrusions formed by migrating cells (Bergert *et al*., 2012; Derivery *et al*., 2008; Langridge and Kay, 2006; Mierke, 2015). These changes in actin structures have been shown to be highly dependent on two key pathways that play compensatory roles and inhibit each other and that regulate the switch between rounded‐amoeboid and elongated‐mesenchymal migratory states (Fig. 1). The activation of Rac1‐WAVE2‐Arp2/3 drives elongated‐mesenchymal adhesive movement, while RhoA/C‐ROCK1/2 pathways drive rounded‐amoeboid migration (Sanz‐Moreno *et al*., 2008; Yamazaki *et al*., 2009) although some degree of Rho‐ROCK‐driven contractility is required also for elongated‐mesenchymal migration (Friedl and Wolf, 2009; Vicente‐Manzanares *et al*., 2009). Cdc42 is required for both elongated‐mesenchymal and rounded‐amoeboid movement depending on engagement of different effectors (Calvo *et al*., 2011; Gadea *et al*., 2008). Interestingly, loss of the Ras regulator RasGRF2 in melanoma cells induces MAT (Calvo *et al*., 2011).

### 6.2 Single‐to‐collective tumour invasion

In fibrosarcoma and breast carcinoma 3D‐spheroids, a spontaneous transition from individual mesenchymal invasion towards multicellular strands (Fig. 1) occurs in follower cells along the microtracks generated by leader cells (Wolf *et al*., 2007). These microtracks are occupied by following coupled cells and therefore, tracks increase in width, ultimately resulting in strand‐like collective invasion (Friedl and Wolf, 2008; Wolf *et al*., 2007).

The microenvironment, in particular ECM porosity, can regulate tumour plasticity and single‐to‐collective transition. Cell jamming is a collective mode of invasion of mesenchymal tumour cells that is imposed by tissue confinement. Dense matrix induces cell–cell interactions, leader–follower cell behaviour and collective migration as an obligate protease‐dependent process (Haeger *et al*., 2014). The conversion to collective invasion with increasing ECM confinement supports the concept of cell jamming as a guiding principle for melanoma and fibrosarcoma cells into dense tissue (Haeger *et al*., 2014; Sadati *et al*., 2013; Vedula *et al*., 2012). In addition, confinement modelled with micropillar arrays can also force collective migration of breast carcinoma cells (Wong *et al*., 2014).

Single‐to‐collective migration can also be induced by gradients or changes in adhesion molecules. For example, when individual cells become attracted by the same chemotactic source, they may first undergo multicellular streaming with short‐lived, dynamic cell–cell junctions. When cell–cell adhesion molecules are then upregulated, the cells may join each other and convert to a collective migration mode (Friedl and Alexander, 2011).

### 6.3 Determinants of plasticity

Determinants of plasticity in cells exhibiting individual mode of migration include physical and molecular cues (proteases, membrane receptors, secreted factors and intracellular signalling pathways), which are broadly highlighted in Figs 3 and 4.

#### 6.3.1 Physical cues

Migration in discontinuous 3D substrates that allow cell–matrix adhesion results in a highly polarized spindle‐shaped morphology in elongated‐mesenchymal cells (Charras and Sahai, 2014; Starke *et al*., 2014; Fig. 3A). However, within discontinuous 3D matrices, if availability of small surface areas for attachment is low, such surfaces might not support adhesion formation and bleb‐based modes of migration are favoured (Petrie *et al*., 2012; Tozluoglu *et al*., 2013).

Another characteristic of ECM is porosity, which determines the confinement of migrating cells. Tissue confinement can also promote single‐to‐collective transitions such as cell jamming (Haeger *et al*., 2014; Sadati *et al*., 2013; Vedula *et al*., 2012). During individual migration, increasing confinement and decreasing adhesion result in increased deformability of the cell and MAT (Liu *et al*., 2015; Tozluoglu *et al*., 2013). The switch in these modes of migration is regulated by a delicate balance between adhesion and actomyosin contractility (Bergert *et al*., 2012).

While the cell cytoplasm is readily deformable in confined conditions, the nucleus is 2–10 times stiffer than the cytoplasm, thus generating a deformability barrier (Wolf *et al*., 2013). The deformability of the nucleus is dependent on the stiffness of nuclear lamina, which is regulated by lamin A/C levels (Lammerding *et al*., 2004, 2006). While low levels of lamins result in increased nuclear deformability, excessive softness of nuclear lamina decreases cell survival. In fact, cancer cells migrating in confined spaces experience nuclear envelope ruptures that result in DNA damage, which is solved using DNA repair machinery and endosomal sorting complexes required for transport (ESCRT; Denais *et al*., 2016; Raab *et al*., 2016). Cancer cells capable of resealing nuclear envelop rapidly could benefit from greater nuclear deformability, increased migration and survival. On the other hand, DNA damage responses induced by reactive oxygen species (ROS) dramatically reduce rounded‐amoeboid invasion *in vitro* and *in vivo*, by suppressing actomyosin contractility (Herraiz *et al*., 2016). In migrating cells, how different types of DNA damage are sensed and repaired will be an important question to solve.

In addition to ECM properties, mechanical perturbations such as interstitial flow can also affect cell migration. In fact, inflammation in cancer can dramatically increase fluid flow between the blood and lymphatic system (Dafni *et al*., 2002; Shieh and Swartz, 2011), causing an increase in migration speed of breast cancer cells (Haessler *et al*., 2012). Interestingly, for breast cancer cells able to migrate using both rounded‐amoeboid and elongated‐mesenchymal motility within 3D collagen type I matrix, interstitial flow favours a switch towards rounded‐amoeboid motility (Huang *et al*., 2015).

#### 6.3.2 Molecular cues

##### 6.3.2.1 Proteases

While pericellular proteolytic inhibition in elongated‐mesenchymal cells drives MAT and cells keep invading (Sahai and Marshall, 2003; Wolf *et al*., 2003), rounded‐amoeboid melanoma cells are able to degrade the matrix (Hooper *et al*., 2006), in some cases even more efficiently than elongated‐mesenchymal melanoma cells (Orgaz *et al*., 2014). This may be due to a higher secretion of certain MMPs such as MMP13 and MMP2. Furthermore, melanoma cells use MMP9 noncatalytic functions to sustain rounded‐amoeboid invasion (Orgaz *et al*., 2014) via regulation of actomyosin contractility.

##### 6.3.2.2 Membrane receptors

Membrane receptors such as β1 integrins can also control migration of individual elongated‐mesenchymal cells (Ahn *et al*., 2012; Friedl, 2004; Wolf *et al*., 2007). Furthermore, CD44 has been shown to be required for individual rounded‐amoeboid invasion (Orgaz *et al*., 2014). CD44 forms a complex with MMP9, which results in the activation of actomyosin contractility in melanoma (Orgaz *et al*., 2014).

##### 6.3.2.3 Secreted factors

Melanoma cells secrete high levels of IL‐6 family cytokines that promote individual rounded‐amoeboid invasion (Sanz‐Moreno *et al*., 2011). HGF receptor Met‐driven signalling has also been implicated in MAT via Rho‐ROCK pathway (Laser‐Azogui *et al*., 2014). Therefore, extracellular ligands govern how integration of signals is achieved in migrating cells travelling through different tumour microenvironments.

##### 6.3.2.4 Intracellular signalling pathways

Actin dynamics determine the type of protrusions. Promotion of actin polymerization in carcinoma cells drives the formation of actin‐rich lamellipodia, whereas blebbing requires both actin polymerization and depolymerization (Bergert *et al*., 2012; Bovellan *et al*., 2014; Derivery *et al*., 2008; Langridge and Kay, 2006; Mierke, 2015).

The balance between antagonistic RhoA and Rac1 signalling determines the mode of migration and lies at the core of tumour cell plasticity in individual migration of several cancer cell types (Sanz‐Moreno *et al*., 2008; Yamazaki *et al*., 2009). Downstream of β3 integrin, adaptor NEDD9 activates Src signalling (involving also p130Cas, Crk) and the Rac GEF DOCK3 (Ahn *et al*., 2012; Carragher *et al*., 2006; Kiyokawa *et al*., 1998; Sanz‐Moreno *et al*., 2008). In turn, active Rac signals through WAVE‐2 promoting Arp2/3‐dependent actin assembly and protrusion formation, driving elongated‐mesenchymal migration (Sanz‐Moreno *et al*., 2008; Yamazaki *et al*., 2009; Fig. 3B). WAVE‐2 suppresses rounded‐amoeboid movement by inhibiting actomyosin contractility (Sanz‐Moreno *et al*., 2008; Yamazaki *et al*., 2009).

Conversely, the Rac‐specific GAPs ARHGAP22 and ARHGAP24 (also known as FilGAP), which are activated by high actomyosin contractility, maintain low levels of Rac activity in rounded‐amoeboid cells (Saito *et al*., 2012; Sanz‐Moreno *et al*., 2008). MAT can be induced through the inhibition of Rac activity (Sanz‐Moreno *et al*., 2008), or indirectly activating Rho by engaging EphA2 (Parri *et al*., 2009). Lowering the levels of RhoA‐negative regulator p27^Kip1^ (Besson *et al*., 2004) also promotes rounded‐amoeboid migration (Berton *et al*., 2009). The antagonistic interplay between Rho‐ROCK and Rnd3 (RhoE) at the cell membrane that regulates blebbing also drives cell plasticity. Absence of PDK1 allows for inhibitory binding of RhoE to ROCK leading to impaired actomyosin contractility and rounded‐amoeboid motility (Pinner and Sahai, 2008b). Importantly, Cdc42 has a dual role as it supports rounded‐amoeboid migration via DOCK10 and the Cdc42 effectors NWASP and PAK2 (Gadea *et al*., 2008). Supporting these data, blocking the Cdc42‐negative regulator and Ras GEF RasGRF2 ablates amoeboid invasion and metastatic colonization (Calvo *et al*., 2011). On the other hand, in elongated‐mesenchymal cells, Cdc42 promotes Rac activity by activating and recruiting ubiquitin ligase SMURF1 to the leading edge via a PAR6–aPKC polarity complex (Osmani *et al*., 2010).

Regulation of protein levels and protein localization drives plasticity. As such, downregulation of SMURF1, which targets RhoA for localized proteasomal degradation in Rac‐dependent protrusions, results in MAT (Sahai *et al*., 2007). Rab5‐dependent endocytosis regulates Rac localization to protrusions supporting therefore elongated‐mesenchymal movement (Palamidessi *et al*., 2008).

##### 6.3.2.5 Transcriptional programmes

While individually invading cells can switch between blebs and protrusions in short timescales (Bergert *et al*., 2012), maintaining cell motility programmes requires a tight temporal coupling of actin dynamics and transcriptional activity (Olson and Nordheim, 2010). Hence, it is no surprise that several transcriptional factors have been implicated in different modes of migration and cellular plasticity. Loss of p53 function via mutant p53 overexpression results in MAT in melanoma cells (Gadea *et al*., 2007). Transcription driven by p53 further suppresses fast rounded‐amoeboid migration via induction of its transcriptional target p53‐induced gene 3 protein (PIG3). PIG3 is an oxidoreductase that produces ROS and further suppresses Rho activity via regulation of ARHGAP5 (Herraiz *et al*., 2016).

In contrast, maintenance of rounded‐amoeboid movement is driven by IL‐6 family of cytokines and the transcription factor STAT3. ROCK can activate JAK/STAT3 signalling generating a positive feedback loop (Sanz‐Moreno *et al*., 2011). As a result of high levels of STAT3 activity, rounded‐amoeboid melanoma cells secrete higher levels of most secreted MMPs (Orgaz *et al*., 2014).

MRTF‐ and SRF‐driven transcription can sustain high actomyosin contractility levels to promote metastasis in melanoma and breast carcinoma cells (Medjkane *et al*., 2009). In breast cancer models, TGF‐β/SMAD induces transcriptional changes that promote a cohesive‐to‐single invasion (Giampieri *et al*., 2009). Those transcriptional changes include genes that control actomyosin contractility (Giampieri *et al*., 2009). In melanoma, TGF‐β promotes rounded‐amoeboid migration, which is perpetuated via SMAD2/CITED1‐mediated transcription of LIF, JAK and the Rho GEF ARHGEF5 (Cantelli *et al*., 2015).

## 7 Therapeutic challenges posed by migratory plasticity

Plasticity or adaptability in terms of cell migration modes likely underlies the failure of some therapies aimed at blocking cancer invasion and metastasis. Several therapies targeting pericellular matrix‐degrading proteases were developed (Coussens *et al*., 2002; Overall and Kleifeld, 2006; Overall and Lopez‐Otin, 2002). However, extensive phase III clinical trials not only failed but even worsened metastatic processes (Coussens *et al*., 2002; Fingleton, 2003; Overall and Lopez‐Otin, 2002; Zucker *et al*., 2000). Such failure was attributed in part to the different roles of specific MMPs (Lopez‐Otin and Matrisian, 2007). However, the MAT that occurs upon pericellular proteolysis inhibition (Friedl, 2004; Sahai and Marshall, 2003; Wolf *et al*., 2003; Wyckoff *et al*., 2006) would add up to the reasons why therapies broadly targeting MMP functions were not successful. In addition, noncatalytic regulation of cell signalling (Orgaz *et al*., 2014) could be an additional reason for the failure of MMP inhibitor‐based therapies (Dufour and Overall, 2013; Overall and Kleifeld, 2006; Zucker *et al*., 2000). Therefore, targeting specific proteolytic and nonproteolytic functions of certain MMPs may provide better results in the clinic.

While targeting MMPs offers a singular therapeutic focal point, it is crucial to keep in mind that the tumour microenvironment presents a heterogeneous and discontinuous environment with varying matrix geometries and degree of stiffness. As a consequence, cells could exhibit MAT spontaneously in response to localized changes in stiffness and this plasticity can impact tumour dissemination *in vivo*. Thus, effective therapies should focus on blocking plasticity by inhibiting multiple intracellular and extracellular drivers of this mode of drug resistance.

## 8 Potential therapeutic targets to block migratory plasticity and tumour cell invasion

Adaptation of cancer cells to different environmental conditions is exemplified by the wide variety of invasion strategies they can adopt. More striking is their ability to switch from one strategy to another to keep on invading. This adaptability is complex, as tumour cell migration plasticity may not need fixed genetic drivers, but it may be aided by accumulated DNA damage in migrating cancer cells.

Such adaptability of cancer cells to change their mode of migration could be considered a type of drug resistance. Therefore, therapies should be aimed at targeting cytoskeletal regulators involved in multiple modes of migration, or combination of drugs aimed at different key targets (Figs 3 and 4). This goes in line with combinational therapies that are currently in clinical trials to stop primary tumour growth. Some key regulators could be β1 integrin, which controls single and collective invasion and the switch from one to another. Several therapeutic interventions are being clinically tested in patients with solid tumours, including peptide ATN‐161, which inhibits binding of α5β1 to fibronectin (Cianfrocca *et al*., 2006; Thundimadathil, 2012), and α5β1‐blocking antibody volociximab (Ricart *et al*., 2008). These therapies are also aimed to block tumour angiogenesis (Cianfrocca *et al*., 2006; Ricart *et al*., 2008).

ROCK lies at the core of cytoskeletal regulation in virtually all modes of migration, therefore appears as a good therapeutic target. Interestingly, a pan‐AGC kinase inhibitor that very effectively targets ROCK (Sadok *et al*., 2015) is being clinically evaluated in advanced solid tumours (ClinicalTrials.gov Identifier: NCT01585701).

Cdc42 or its effectors could be also suitable candidates given their involvement of both rounded‐amoeboid and elongated‐mesenchymal invasion strategies. A combination of drugs targeting cell adhesion and the actomyosin core machinery could also be considered.

Furthermore, careful attention should be given to targeting transcriptional programmes that self‐perpetuate invasion strategies (JAK/STAT3, TGF‐β/SMAD) and control processes such as tumour promoting inflammation and immunosuppression. Given the protumorigenic roles of the JAK/STAT3 pathway, inhibition of JAK/STAT3 in solid tumours is currently being evaluated (Buchert *et al*., 2016). Moreover, several inhibitors of the TGF‐β pathway are being developed and clinically tested for a number of cancers (Neuzillet *et al*., 2015). However, the dual role of TGF‐β as tumour suppressor or prometastatic (Massague, 2008) anticipates that targeting its transcriptional targets and/or regulators might be a better approach to block only its prometastatic effects.

## 9 Concluding remarks

Tumour cells usually encounter heterogeneous and discontinuous microenvironments. As a consequence, cancer cells need to adapt spontaneously in response to localized physical and chemical changes. The minimum machinery required to drive all different types of migration comprises the actomyosin cytoskeleton. Differential regulation of actomyosin machinery is what drives plasticity and different modes of migration, blockade of which is essential to prevent cancer invasion and metastasis. Thus, future therapies for preventing metastasis should focus on selective pharmacological inhibition of actomyosin machinery within cancer cells.
